# *Suffix*-specific RNAi Leads to Silencing of F Element in *Drosophila melanogaster*

**DOI:** 10.1371/journal.pone.0000476

**Published:** 2007-05-30

**Authors:** Nickolai A. Tchurikov, Olga V. Kretova

**Affiliations:** Engelhardt Institute of Molecular Biology, Russian Academy of Sciences, Moscow, Russia; Netherlands Cancer Institute, Netherlands

## Abstract

Separate conserved copies of *suffix*, a short interspersed *Drosophila* retroelement (SINE), and also divergent copies in the 3′ untranslated regions of the three genes, have already been described. *Suffix* has also been identified on the 3′ end of the *Drosophila* non-LTR F element, where it forms the last conserved domain of the reverse transcriptase (RT). In our current study, we show that the separate copies of *suffix* are far more actively transcribed than their counterparts on the F element. Transcripts from both strands of *suffix* are present in RNA preparations during all stages of *Drosophila* development, providing the potential for the formation of double-stranded RNA and the initiation of RNA interference (RNAi). Using *in situ* RNA hybridization analysis, we have detected the expression of both sense and antisense *suffix* transcripts in germinal cells. These sense and antisense transcripts are colocalized in the primary spermatocytes and in the cytoplasm of the nurse cells, suggesting that they form double-stranded RNA. We performed further analyses of *suffix*-specific small RNAs using northern blotting and SI nuclease protection assays. Among the total RNA preparations isolated from embryos, larvae, pupae and flies, *suffix*-specific small interfering RNAs (siRNAs) were detected only in pupae. In wild type ovaries, both the siRNAs and longer *suffix*-specific Piwi-interacting RNAs (piRNAs) were observed, whereas in ovaries of the Dicer-2 mutant, only piRNAs were detected. We further found by 3′ RACE that in pupae and ovaries, F element transcripts lacking the *suffix* sequence are also present. Our data provide direct evidence that *suffix*-specific RNAi leads to the silencing of the relative LINE (long interspersed element), F element, and suggests that SINE-specific RNA interference could potentially downregulate a set of genes possessing SINE stretches in their 5′ or 3′ non-coding regions. These data also suggest that double stranded RNAs possessing *suffix* are processed by both RNAi and an additional silencing mechanism.

## Introduction

Retroelements are ancient components of the genome, and are potential participants in some RNA-related regulatory mechanisms in the cell. The recent discovery of RNAi has extended our knowledge of such processes by uncovering mechanisms in which short RNA molecules are used by protein complexes for the recognition of specific nucleotide sequences that are important for the regulation of gene expression and also the formation of chromosomal structures [1]. In a landmark paper by Fire and colleagues [2], it was demonstrated that double-stranded RNA (dsRNA) is the trigger for RNAi silencing mechanisms. A number of mechanisms were subsequently described in which control of mRNA translation, the formation of heterochromatin structures, and the silencing of either mobile elements or unpaired DNA is mediated by RNAs as universal intermediates in homology sensing [3]–[5]. In some of these mechanisms, it has been postulated that ubiquitous retroelements could serve not only as targets for silencing, but also as tools that provide RNA sequences for regulation.

Retroposition is an ancient genetic mechanism underlying the flow of information from RNA to DNA, resulting in the appearance of new copies of a corresponding sequence in the genome. Several classes of retroelements have now been detected during the last few decades: non-LTR mobile elements (or LINEs), LTR-elements that are closely related to retroviruses, and short retroelements (or SINEs). SINEs are too small to harbor a coding function, and for their transposition they use reverse transcriptases encoded by LINEs. Until now, the major portion of the SINEs described in different genomes are derived from either small structured RNA molecules of tRNAs or from 7SL RNA, which forms part of the ribosomal complex [6] and has an internal RNA polymerase III promoter [7]. Studies indicate that the internal promoter is not sufficient for *in vivo* transcription of a SINE, and that some control signals are required from the insertion site [8]. Hence, the majority of the SINE copies are transcriptionally inactive, i.e. non-functional fossil relics with respect to retropositioning [9]. Without selective pressure, they accumulate mutations or decay over the course of evolution. It is possible that a small part, or even a particular SINE copy (master or source gene), could be transcribed and its RNA potentially used for retropositioning [6], [10]. In addition, although the mechanisms underlying retroposition remain unclear, several factors have been suggested to be important including the ability of the specific transcript to compete for association with the enzymatic machinery “borrowed” from LINEs for mobilization; and the length and homogeneity of the poly(A) stretch, which allows for effective priming [11]. The discovery of RNAi mechanisms, which are considered to be not only an ancient protective mechanism against retroelements, but are also regarded as a physiological tool for the regulation of gene activity [12]–[14], has made the study of transcription patterns of different retroelements more significant.

*Suffix* is an unusual example of a short retroelement. Although it has a poly(A) stretch and a size that is typical of a SINE, it lacks the usual RNA polymerase III promoter and possesses a short open reading frame. Previously, *suffix* was found as a separate repetitive element with different sequences around (separate copies), as well as on the extreme 3′ ends of some genes and also on the 3′ ends of F and Doc elements [15]–[17]. Comparison of sequences of *suffix* and F elements led to the first demonstration that SINEs and LINEs share a common 3′ sequence, possessing a small region of coding sequence, a poly(A) signal and a poly(A) site [16], [18]. More recently, new examples of several pairs of SINEs and LINEs from vertebrates and plants have been described [9], [19].

It has recently been reported that in the *Drosophila* germline there are repeat-associated small interfering RNAs (rasiRNAs) that protect against retroelements by a novel RNA silencing mechanism [20]. These RNAs are distinguishable from siRNAs by their longer length (24–29 nucleotides, nt) and by the lack of the 2′,3′ hydroxyl termini that are characteristic of miRNAs and siRNAs. Hence, silencing mechanisms involving rasiRNAs are distinct from the earlier described RNAi and miRNA pathways. In addition, they do not require Dicer-1 or Dicer-2 RNases and function through the PIWI protein family (Aub, Piwi, and Ago3). RNAs of 29–30 nt from testes that interact with Piwi proteins have also been described in mammals [21] and are known as piRNAs.

In our present study, we show that sense and antisense *suffix* transcripts are present during all stages of *Drosophila* development and are co-localized in the germline. However, *suffix*-specific siRNAs, the putative RNAi products, are detectable only in pupae. It is of interest that in the wild type ovaries, two classes of small *suffix*-specific small RNAs are present, siRNAs and piRNAs, as this suggests that an additional silencing mechanism targeting the *suffix*-containing transcripts is involved in the germ line. F element transcripts lacking the *suffix* stretch can also be detected in pupae. These data indicate that the *suffix* element is involved in developmentally regulated RNA-interference, which leads to silencing of the F element. Our current data also suggest a hypothetical novel mechanism, whereby the concerted silencing of genes occurs by RNAi targeting of a SINE sequence in the non-coding regions of mRNA sequences.

## Results

### Transcriptional patterns of *suffix* and F elements

**Figure 1:**
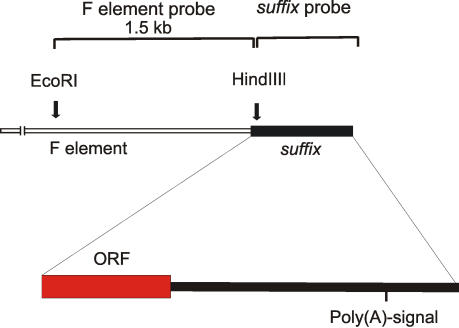
Relationship between the *Drosophila* F element and *suffix.* Neighboring DNA fragments (shown in brackets) were used for the preparation of both *suffix*-and F element-specific RNA probes for Northern analysis.

**Figure 2:**
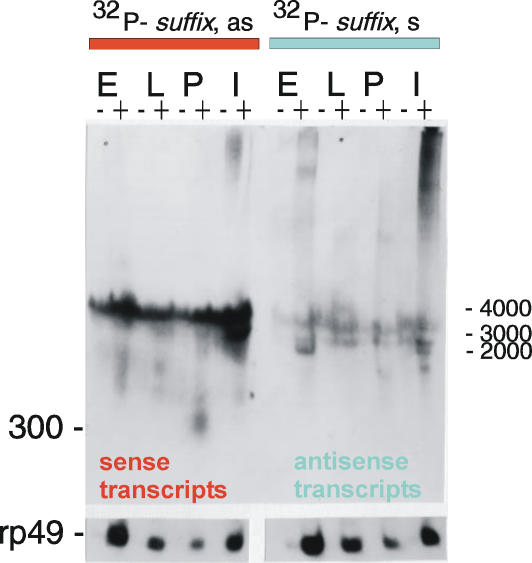
Northern blot analysis of *suffix*-specific transcripts. Hybridizations were performed with *suffix*-specific antisense (as) or sense (s) [^32^P]-labeled RNA probes. The lanes with poly(A)^+^ and poly(A)^−^ RNA samples, isolated from *Drosophila* embryos (E), larvae (L), pupae (P) and imago (I) are shown. Positions of the RNA markers in nt are shown on the right. The band of around 300 nt in length is shown on the left. Quantitation of the RNA content by hybridization with an rp49-specific probe is shown at the bottom.

Previously, we demonstrated that *suffix* elements found in genes are present in a reversed polarity, and that the poly(T)-containing strand (minus strand) forms the last, very short intron and exon [16]. To further study the transcripts corresponding to both strands of this element in more detail, we performed Northern blotting analysis under stringent hybridization conditions. In these experiments, the signals should come from transcribed *suffix* sequences within genes, from F elements and also from transcripts of separate individual *suffix* copies. In our preliminary experiments, we found that under such conditions, signals from divergent *suffix* copies and Doc-like versions of this element could not be detected. Figure 1 depicts the neighboring regions of the F element that were used for the preparation of the strand-specific [^32^P]-RNA probes. For the preparation of these probes, we used *suffix* sequences and the upstream sequence of F elements subcloned into pGEM-vectors. The *suffix*-specific antisense [^32^P]-RNA probe would be expected to detect sense, poly(A)-containing transcripts generated from separate copies of *suffix* and the F element. We observed a major component of *suffix*-containing RNA bands (approximately 3500 nt) in both poly(A)^+^ and poly(A)^−^ RNA samples during all developmental stages (Figure 2). Careful analysis revealed that this band was located above the smaller 18S rRNA and fragments of 28S RNA. The current databases contain one full-length, 3.6 kb cDNA (AC:AY71740), harboring the *suffix* element in the 5′ non-coding sequence. This cDNA has a coding region specifying a reverse transcriptase that is homologous to the *pilger* element, a non-LTR *Drosophila* retrotransposon (AC:AJ278684).

It has been reported that *Alu* sequences are also detectable in both poly(A)^+^ and poly(A)^−^ RNA preparations [11]. Interestingly, only in the *Drosophila* line under study (Oregon-Shostak), and not in several other lines that we tested throughout our analyses, did we observe a short poly(A)^+^ transcript of about 300 nt in length, which is the size expected for a *suffix* full-length transcript. Moreover, this transcript was detected only in pupae. There are weaker transcripts of different lengths containing the *suffix* sense strand in poly(A)^+^ RNA preparations in embryos, larvae, pupae and flies. Our data on the nature of *suffix*-containing transcripts obtained with RLM-RACE (Ambion), will be described in a separate report.

A [^32^P]-sense-RNA probe reveals the presence of *suffix* antisense transcripts that would be predicted to come mainly from copies of these elements within genes. As expected, these transcripts are more prominent in poly(A)^+^ samples and their pattern and intensity changes during development. Some bands may also correspond to antisense transcripts generated from F element copies.

**Figure 3:**
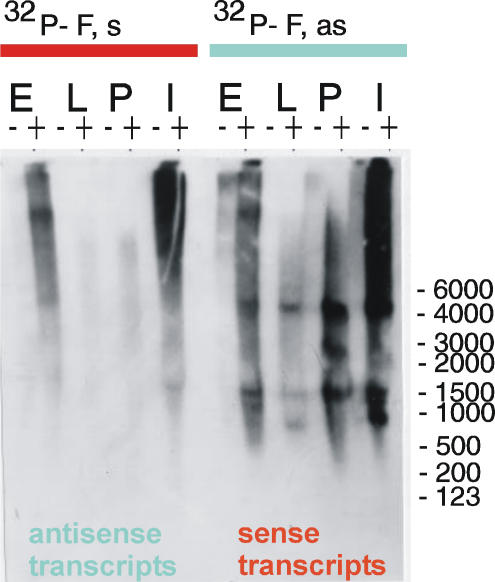
Northern blot analysis of F element-specific transcripts. Hybridizations were performed with F element-specific antisense or sense [^32^P]-labeled RNA probes. The labeling is as described for Figure 2.

The same blots were re-hybridized with F element-specific [^32^P]-RNA probes after stripping of the previous probe. It has been believed for some time that the F element is transcribed for a short duration, and then only in embryos [22]. Northern analysis of F element is also hampered by the presence of very long transcripts in embryos, pupae and imago that cause smearing from the top of the gel [23]. However, modern techniques allow us to observe the transcription patterns of F element in more detail. In our current experiments, we observed a more discrete picture under stringent conditions of hybridization and washing. A strong 4700 nt band, corresponding to the full-length F element transcript [22], and a number of smaller poly(A)^+^ bands were revealed by the use of an F element-specific antisense probe in embryos, larvae, pupae and flies (Figure 3, right panel).

*Suffix* sequences should be present at the 3′ ends of all poly(A)^+^ F element transcripts, but the corresponding bands are not visible against a background of much more highly abundant RNA molecules generated by the independent *suffix* copies (compare the left panel in Figure 2 with the right panel in Figure 3). Although the neighboring fragments of the F element were used for the preparation of the *suffix*- and F element-specific probes, it is clear that the patterns of hybridization for the antisense [^32^P]-probes corresponding to both the *suffix* and F elements are very different. This fact demonstrates that *suffix* is transcriptionally very independent from the F elements and that the signals generated by the separate *suffix* copies are much higher. Poly(A)-containing sense transcripts corresponding to F elements were also observed on a *suffix*-probed blot but only after a longer exposure (data not shown). Another difference is that, in the case of F elements, both sense and antisense transcripts are mainly polyadenylated. This again demonstrates that the essential portion of the *suffix*-specific transcripts is not generated from the F element.

There are known to be very long polyadenylated transcripts possessing F element and *suffix* sequences in embryos and flies, which probably come from regions of heterochromatin where the majority of these copies are found [15], [22]. Both elements have another feature in common–their transcripts are generated from both strands. In addition, whereas, symmetrical *suffix* transcripts are present during all stages of development, symmetrical polyadenylated transcripts from F elements are present mainly in embryos and flies. In the current *Drosophila melanogaster* databases, there are only 8 *suffix*-containing ESTs that correspond to F elements. Hence, our present data on transcription patterns of *suffix* clearly indicate that the databases are still poor in *suffix*-containing transcripts.

### Sense and antisense transcripts of both *suffix* and F element are located in the same germline cells

**Figure 4:**
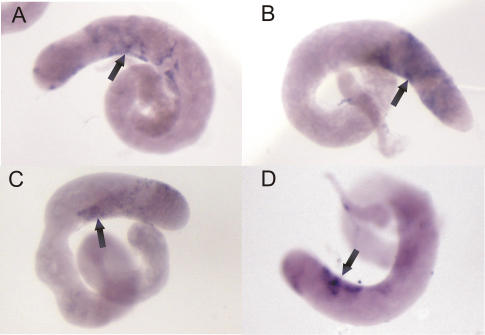
Expression patterns of *suffix* and F elements in *Drosophila* testis. *In situ* hybridizations with DIG-labeled, strand-specific RNA probes were performed (Materials Methods). (A, B) The patterns revealed by *suffix* sense and antisense probes, respectively. (C, D) The patterns revealed by F element sense and antisense probes, respectively. Arrows indicate the transcripts detected in primary spermatocytes.

To investigate the possibility that dsRNA may be formed by sense and antisense RNAs coming from both *suffix* and F element, we tested whether these transcripts are expressed in the same cells. We selected testes and ovaries for *in situ* hybridization analysis using the same RNA probes that were used in our Northern blots, but this time labeled with DIG (see Materials and Methods). We found that the *suffix* probes hybridized in the nuclei of primary spermatocytes (Figure 4A,B) and that F element probes revealed this same pattern of hybridization in testis (Figure 4C,D). It has been demonstrated that primary spermatocytes are derived from the primary spermatogonial cells by four mitotic divisions that produce 16 primary spermatocytes in the cyst [24].

**Figure 5:**
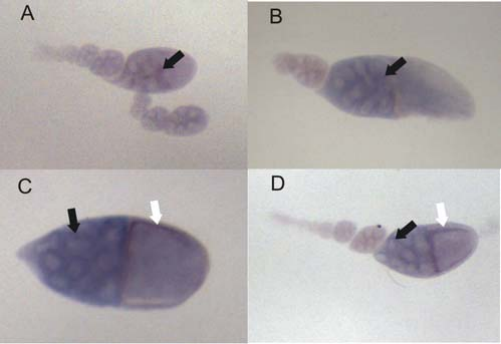
Expression patterns of *suffix* and F elements in *Drosophila menalogaster* ovaries. *In situ* hybridizations with DIG-labeled, strand-specific RNA probes were performed (Materials Methods). (A, B) The patterns revealed by *suffix* sense and antisense probes, respectively. (C, D) The patterns revealed by F element sense and antisense probes, respectively. Black arrows indicate the transcripts detected in the cytoplasm of nurse cells. White arrows denote F element transcripts in follicle cells.

In the mature egg chamber of the ovaries, which consists of the oocyte and nurse cells that are surrounded by somatically derived follicle cells, we detected sense and antisense transcripts of *suffix* in the cytoplasm of the nurse cells (Figure 5A,B). The F element probes were also found to hybridize in the cytoplasm of the nurse cells, but they also reveal the presence of transcripts in the follicle cells (Figure 5C,D). Again, the same patterns were observed for both sense and antisense probes. Although *suffix* and F element patterns in ovaries have one obvious difference, the patterns revealed by sense and antisense probes for each element were found to be consistent.

It has been postulated that if sense and antisense RNAs are present in the same cell, they can form dsRNA, but it is difficult directly check for the formation of dsRNA *in vivo*. Our *in situ* hybridization data from two *Drosophila* organs indicate that sense and antisense RNAs generated from *suffix* or F elements are present in the same cells. Hence, there is a potential for the formation of the corresponding dsRNA, at least in some tissues and organs.

### *Suffix* and RNA silencing mechanisms

**Figure 6:**
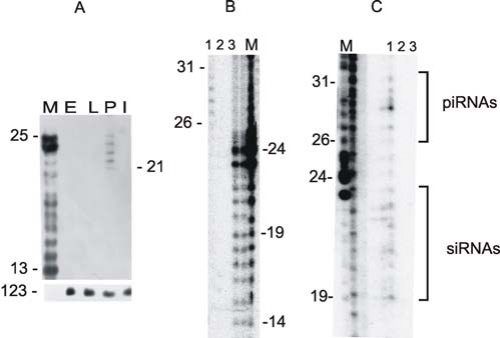
Analysis of *suffix*-specific small RNAs by Northern blot analysis or SI nuclease protection assay. Numbers indicate the lengths of the RNA molecules in nt. M indicates RNA markers, containing a mixture of intact 25 and 14 nt RNAs and a fragmented 25 nt or 86 nt RNAs (see Materials and Methods). (A) Blots containing fractionated total RNA samples isolated from embryos (E), larvae (L), pupae (P) and imago (I), were probed by *suffix*-specific antisense [^32^P]-labeled RNA probes (see Figure 1). The same results were obtained with an identical blot after hybridization with a *suffix*-specific sense [^32^P]-labeled RNA probe (not shown). The total *Drosophila* RNA content on lanes is validated by hybridization with a 123 nt 5.8S RNA-specific probe (see Materials and Methods). (B) SI nuclease protection assay of total *Drosophila* RNA isolated from *dcr^2^/dcr^2^* ovaries (Materials and Methods). [^32^P]-labeled 86 nt antisense RNA probe corresponding to 5′ region of the *suffix* was hybridized overnight with 5 µg of total RNA (1) or with 5 µg of yeast tRNA (3). After SI nuclease digestion at 20°C the probes were separated on 12% denaturing polyacrylamide gel. 2–result obtained with 5 µg of total *Drosophila* RNA without hybridization (overnight incubation at 0°C). M–RNA marker. (C) SI nuclease protection assay of total *Drosophila* RNA isolated from the wild type ovaries. Indications are the same as in (B). Regions corresponding to siRNAs and piRNAs are shown in brackets.

The presence of sense and antisense transcripts of different lengths generated from *suffix* sequences during all stages of *Drosophila* development, and the fact that these transcripts might be expressed in the same cells, provides the potential for forming dsRNA species *in vivo*, and the triggering of RNAi mechanisms leading to sequence-specific degradation of the cognate RNAs [12]–[14]. To test whether this is the case, we examined the presence of *suffix*-specific siRNAs in the total RNA samples isolated from embryos, larvae, pupae and imago. Figure 6A shows that siRNAs ranging in length from 21 to 25 nt are observed only in pupae. This result is not due to higher amounts of pupal RNA in the lane, which was tested using 5.8S ribosomal RNA (Figure 6A). These data are also reproducible, as the same results were observed in three experiments with different RNA preparations. Thus, although symmetrical transcription can be observed throughout development, *suffix*-specific siRNAs are observed during only one particular stage. Of course, we cannot exclude the possibility that smaller quantities of these siRNAs are below the threshold of detection for this method.

To determine whether Dicer is involved in the formation of the detectable 21–25 nt siRNAs, we analyzed total RNA preparations from ovaries of *dcr^2^/dcr^2^* flies [25]. Using an SI nuclease protection assay, we observed only the longer 26–31 nt small RNAs (Figure 6B). These data clearly demonstrate that Dicer-2 is required for formation of *suffix*-specific siRNAs. This experiment also drew our attention to the recently detected 24–29 nt small RNAs formed from the transcripts of some retroelements and repetitive elements [20]. These transcripts are not processed into siRNAs in the germ line, but are converted by Dicer-1 and Dicer-2 independent pathways into 24–29 nt rasiRNAs. It is also noteworthy that in the wild type ovaries we detected the formation of both *suffix*-specific 19–25 nt siRNAs and longer 26–31 rasiRNAs or piRNAs (Figure 6C). This indicates that in the ovaries of the Dicer-2 mutant, only one degradation pathway for *suffix*-containing transcripts is affected, whereas an additional pathway that produces longer RNAs remains active. It follows from these findings therefore that in the germ line, *suffix*-containing RNAs are controlled by two distinct silencing mechanisms using siRNAs and piRNAs.

*Suffix* is located on the very end of the F element and supplies it with its last conserved RT domain, polyadenylation signal and site [16], [18]. For this reason, the degradation of the *suffix* region in F element mRNA should lead to the silencing of this LINE during the pupal stage of development, at least in some tissues, or in ovaries.

### Analysis of the 3′ ends of F element transcripts

**Figure 7:**
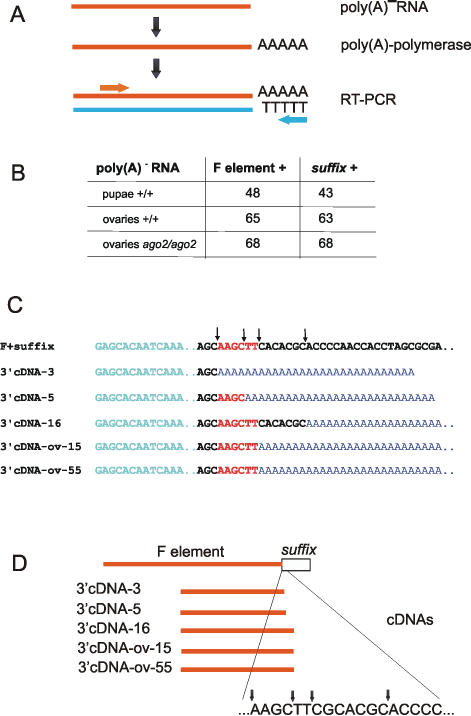
Cloning of F element transcripts lacking *suffix* sequences using 3′RACE. (A) Schematic illustration of the procedures used (see Materials and Methods). After the addition of a poly(A) tail to the poly(A)^−^RNA preparations isolated from pupae or ovaries, the samples were used for reverse transcription with a poly(T) primer and subsequent PCR using poly(T)-and F element-specific primers. (B) The number of clones identified by hybridization with *suffix*-specific or F-element-specific probes in colony-hybridization experiments. Five clones from pupae (3, 5, 16, 28 and 38) showed hybridization with the F-element probe only were selected for sequencing. Similarly, two clones (15 and 55) were isolated from the wild type ovaries. (C) The sequences of the cDNA clones isolated from pupae or ovaries that were truncated by RNA silencing mechanisms at the very beginning of the *suffix* sequence (shown in bold) are presented (clones 38 and 28 are identical by sequence to clones 5 and 16, respectively). (D) Diagram showing the positions of the cut sites at the very beginning of the *suffix* sequence in the cDNA clones corresponding to F element transcripts, indicated by arrows.

We employed 3′ RACE to test whether *Drosophila* pupae contain F element transcripts lacking the *suffix* region as a result of *suffix*-specific RNA silencing. This approach and the results are illustrated in Figure 7A. Poly(A) polymerase was used for the addition of poly(A) tails to poly(A)^−^ RNA molecules. After PCR amplification using an F element-specific primer and poly(T), three unique cDNA clones binding only with an F element-specific probe were isolated by colony hybridization (Figure 7B). This probe corresponds to F element sequences located just downstream from the specific primer used. The sequences of the clones are shown in Figure 7C. Excised sites in the clones are located at the very beginning of *suffix* in F element mRNA (Figure 7C,D). The same method was used for the isolation of 3′ ends of F element transcripts using total RNA preparations from embryos, larvae and imago. Only F element mRNAs possessing *suffix* sequence were obtained (not shown). We also used 3′ RACE to test whether in *Drosophila* ovaries, where both siRNAs and piRNAs are detectable, there are F element transcripts lacking the *suffix* region. We isolated two clones (cDNA-ov-15 and cDNA-ov-55) containing the same cut site at the 5′ end of the *suffix* region in the F element mRNA (Figure 7B–D). A similar procedure, designed for the isolation of 3′ ends of F element transcripts without *suffix* sequences, was performed using poly(A)^−^ RNA from ovaries of *ago2^414^/ago2^414^* mutant [26]. In this case however, only F element transcripts possessing *suffix* sequences were obtained (Figure 7B). These data indicate that the levels of 3′ truncated F element transcripts are reduced in the Ago2 mutant. Probably the major portion of 3′ truncated F element mRNAs comes from RNAi mechanism. Really, the quantitation of the nuclease protection data (lane 1, Figure 6C) shows that about 60% of the detected small RNAs correspond to siRNAs. Taken together, these results indicate that in some tissues in pupae and in ovaries, *suffix*-specific RNA silencing mechanisms are initiated and result in the appearance of 3′ truncated F element RNAs lacking *suffix* sequence possessing the last RT domain. We thus conclude that the observed cut sites in the F element transcripts do not correspond to non-specific degradation, but are produced by RNA silencing mechanisms.

## Discussion

We have observed a complex pattern of somewhat long transcripts generated from both strands of *suffix* and from F elements in *Drosophila*. Northern blotting and *in situ* hybridization analysis indicate that *suffix* transcription is highly complex and is regulated during development. *Suffix* is mainly transcribed from different sites of insertion as a component of longer RNA molecules, and only a small portion of these correspond to F elements. Separate *suffix* copies are also much more actively transcribed that those residing in F elements. Our data indicate that separate *suffix* copies are transcribed independently of the F element, but that the transcription of both elements is regulated coordinately. Both elements are expressed in testis and ovaries in the same cells, with one exception. Interestingly, both sense and antisense transcripts of both elements are expressed in the same cells.

Both sense and antisense *suffix* transcripts were found in total RNA preparations during all developmental stages of *Drosophila*. Nevertheless, our data on siRNA detection and 3′RACE cloning strongly suggest that, at least in some cells in the pupae, *suffix*-containing RNAs are involved in RNAi mechanisms. We thus conclude that *suffix*-containing transcripts form dsRNAs that trigger RNAi. Degradation of the 3′ end in F element transcripts, where *suffix* sequences are located, removes part of the coding region, and also the polyadenylation signal and polyadenylation site, and this will necessarily cause silencing of the F element. *Suffix* is therefore likely to play a role in the regulation of F element expression in some tissues and organs of pupae, which is why we did not observe the degradation of all *suffix*-containing transcripts isolated from the whole body. We speculate that in some cells *suffix* serves as a tool for a silencing of the F element.

In previous studies, *suffix* sequences were found at 3′ ends in the ribosomal protein L36A (CG208) and in the pOT2 (CG363) genes [16]. In these genes, the *suffix* regions possess a functional polyadenylation signal and site. It follows therefore that *suffix*-specific siRNAs could potentially target the corresponding transcripts and give rise to silencing of these genes in some cell types. It is possible also that other genes containing this element in their 5′ or 3′ non-coding sequences are regulated in the same way.

The transcription of both *suffix* strands and the degradation of its transcripts by RNA silencing mechanisms are developmentally regulated. The former gives different patterns of element transcription in development, while the latter leads to siRNA formation, at least during the pupal stages of development, although *suffix* sense and antisense RNAs are even more abundant in embryos and during the imago stages. It is conceivable that RNAi mechanisms involving these sequences are downregulated in particular organs and tissues during development by as yet unknown factors. In this case, the formation of dsRNA alone is not sufficient to trigger RNAi. Recently, it was shown in *Drosophila* that Dicer-2 is not required for the formation of *roo* rasiRNA [20]. On the other hand, it was also demonstrated that the overexpression of downstream Argonaute proteins in *C. elegans* enhances silencing, suggesting that some proteins are limiting for RNAi [27]. It is possible that some tissues and organs in pupae are limited for particular proteins involved in RNAi, and the presence of these proteins are needed for this process to be initiated. Intriguingly, the sizes of the *suffix*-specific siRNAs were found to be between 21–25 nt, whereas LTR retroelements, such as *roo, mdg1*, and *gypsy*, non-LTR *I* element and *Het-A* rasiRNAs are about 24–29 nt long [20]. It has been shown, however, that Dicer-2-dependent siRNAs are produced with a periodicity of 22 nt [28]. Moreover, the analysis of *Su(Ste)* rasiRNAs has revealed little or no periodicity in processing of its long dsRNA triggers [20]. This may be true also for the *suffix*-specific RNA silencing mechanisms, as we have observed three excision sites in F element transcripts separated by distances of 4, 6, 9 and 13 nt (Figure 7).

Only one class of small RNAs, 24–29 nt long rasiRNAs or piRNAs, corresponding to a number of *Drosophila* retroelements, have been detected previously in the *Drosophila* germline [20] . In our present study of *Drosophila* ovaries, we detected two classes of *suffix*-specific small RNAs: 21–25 nt long siRNAs and 26–31 nt long piRNAs. In ovaries of the Dicer-2 mutant, we detected only 26–31 nt long piRNAs, whereas 19–25 nt long siRNAs were also detected in the wild type ovaries. These data indicate that the *suffix* containing RNAs in the germ line are processed in at least two ways, i.e. by production of 21–25 nt long siRNAs (Ago-pathway) and by production of 26–31 nt piRNAs (Piwi-pathway). We thus speculate that the short *suffix* dsRNAs formed by sense and antisense transcripts of the element itself, and their hybrids with longer RNAs, can be processed in the germline in different ways. This difference between *suffix* RNA silencing and the silencing of other retroelements might be determined by the unusual behavior of *suffix*. Although *suffix* is mostly located in heterochromatic regions as a separate element or as a part of F element, it is also detectable in the opposite polarity at the very 3′ end of some genes [16]. In heterochromatin, *suffix* has also been found in the CAACA-microsatellite or amongst regions of different retroelements [29]. Our data indicate that different silencing mechanisms may be involved in *Drosophila* to protect this organism from the expression of different retroelements.

We observed the coordinated expression of *suffix* and the F element in the cytoplasm of the nurse cells in ovaries. The only evident difference between both elements is that the F element is also transcribed in the follicle cells located around the oocyte. Analysis of the expression of these elements in different RNA silencing mutants is now underway in our laboratory.

Because we observed that sense and antisense *suffix* and F element transcripts can be present in the same cells of the germline, it follows that mechanisms exist that control transcription from both strands of both elements in the same cells. Recently, it was reported that non-LTR retroelements can avoid accidental integration by interacting with a target-specific transcription factor to direct its integration [30]. *Suffix* possesses the 3′ end region required for the recognition of RT specified by the F element. *Suffix* also uses enzymes provided by F elements for its integration and its short transcripts likely integrate at the same sites as F element transcripts. In this case, the same pattern of distribution of transcripts would be expected for both elements. The integration of *suffix* copies in both orientations could give rise to sense and antisense transcripts in the same cells. Our current data on the distribution of *suffix* and F element sense and antisense transcripts in the germline support this contention.

**Figure 8:**
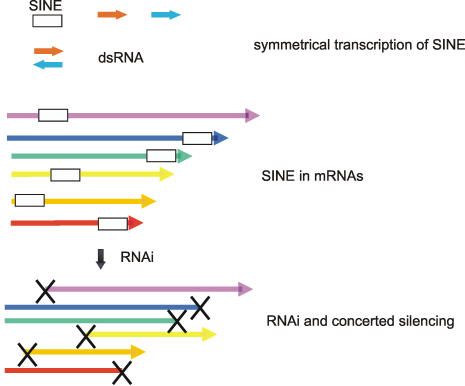
Hypothetical model for the concerted regulation of genes containing a SINE in their 5′ or 3′ non coding regions. Symmetrical transcription of a SINE sequence, co-transcribed as part of a number of larger RNA molecules, leads to the formation of dsRNA and thus to RNAi. As a result, SINE-specific siRNA molecules target the SINE-containing mRNAs, leading to the cleavage of the 5′ or 3′ non coding stretches that are important for gene expression and thus result in the concerted silencing of the SINE-containing genes.

One possible reason for the formation of *suffix*-specific siRNAs and piRNAs is that during the pupal stages of *Drosophila* development or in the germline, abnormal *suffix*-containing transcripts are abolished, and mechanisms that protect against retroelement-containing RNAs are then switched on. Another potentially more interesting possibility, however, is that the dispersion of SINEs within transcripts provides a mechanism for concerted gene-silencing. Based on this speculation, SINEs could be considered not as selfish components of the genome, but as elements of biological significance that have important functions at the RNA level as components of RNAi. In this way, the degradation of the regions important for expression of a specific set of genes possessing particular SINE elements in 5′ or 3′ non-coding regions could be achieved (Figure 8).

*Suffix* has a functional coding sequence that is part of the F element at the protein level. Moreover, it functions also at the RNA level, since it is involved in RNA-regulation. The protein domain specified by *suffix* within the F element forms an 8^th^ conserved domain of the reverse transcriptase [31]. The other 7 conserved domains have been described for different retroelements [32]. Some specific groups of LINEs, including F elements, have additional ancient RT domains (Kretova and Tchurikov, unpublished). Evolutionary pressure on *suffix* could act upon both the RT and RNA-regulation functions of the element. Both mechanisms–reverse transcription and RNAi–are considered ancient and evolutionary conserved mechanisms for the synthesis of DNA copies on RNA templates, and for the regulation of the expression of host or foreign RNAs. The function of *suffix* as a conserved SINE remains unknown however and as a separate element, *suffix* is clearly unable to serve as a coding sequence. There are some data on the role of SINEs in stress defense and in the post-transcriptional stimulation of expression of different mRNAs [7], [33], [34]. Our present experiments with heat shock-treated flies did not detect any change in the *suffix* transcription pattern (data not shown).

Separate *suffix* copies could originate from the 3′ end of the F element. Recently, we identified a weak internal promoter spanning the junction between the F element and *suffix* that is active in cultured cells (Tchurikov and Kretova, unpublished). Thus, the F element could be a source gene for *suffix* and provide it with 3′end sequence recognized by RT and other enzymes associated with retroposition. In return, *suffix* may downregulate F elements by RNA silencing mechanisms, which probably allow this LINE/SINE family to replicate without killing the host.

## Materials and Methods

### Northern-blot analysis

Approximately 20 µg of poly(A)^+^ or poly(A)^−^ RNA samples isolated from the Oregon-Shostak line were electrophoresed in 2 mm thick, 1.2% agarose gels containing 25 mM NaPO_4_ (pH 7), 0.5 mM EDTA and 5% formaldehyde and blotted in 20×SSC onto Hybond-N+. Hybridization was performed in a solution containing 50% formamide, 5×SSC, ficoll, polyvinylpyrolidone, BSA, and SDS all at a concentration of 0.1%, denatured salmon DNA (50 µg/ml), tRNA (50 µg/ml) and 5-10×10^6^ cpm of the appropriate probe. 10^9^ cpm/µg of the corresponding RNA probes were synthesized with T7 RNA polymerase *in vitro* using adjacent fragments of F element-containing clones as shown in Figure 1. These corresponding fragments had been subcloned into either pGEM-1 or pGEM-2 vectors. The final plasmids were linearized completely with EcoRI or HindIII enzymes, extracted with phenol/chloroform, precipitated with ethanol, washed three times with 70% ethanol-0.1M NaCl, dried, and then diluted in 0.1xTE to a final concentration of 1 µg/µl. RNA synthesis was performed in 20 µl of solution containing 1 µg of DNA template, 40 mM Tris-HCl (pH 7.5), 6 mM MgCl_2_, 2 mM spermidine, 10 mM NaCl, 10 mM DTT, 1 u/µl RNasin, ATP, GTP, CTP (500 mM each), 20–40 µCi [α-^32^P] UTP (6000 Ci/mmol, EIMB) and 20 u of T7 RNA polymerase (Fermentas).

The RNA probes were added to the hybridization mixture following 24 h of pre-hybridization at 43°C. After further hybridization for 48 h at 43°C with the relevant probes, the membrane was washed twice (20 min each) in a 2×SSC, 0.1% SDS solution at 43°C, then 3 times at 65°C in the same solution and finally twice again at 65°C in 0.2×SSC, 0.1% SDS. 6000-200 nucleotide (nt) RNA markers were purchased from “Pequlab”, Erlangen, Germany.

### Study of siRNAs

Approximately 20 µg of a total RNA preparation was subjected to elecrophoresis on a 15% denaturing, 1 mm thick, polyacrylamide gel. The gel was then washed twice in distilled water for 5 min each (removing the urea), and then stained twice in 0.1 M ammonium acetate-ethidium bromide for 15 min each and photographed. The gel was placed in a 0.5×TBE solution and electroblotted in this solution onto Hybond N+ for 30 min (300 v/10 cm). Hybridization was performed in 25% formamide, 0.5 M NaCl, 0.1 M sodium-phosphate buffer, 25 mM EDTA; ficoll, polyvinylpyrolidone, and BSA (0.1% each), 0.1% SDS, denatured salmon DNA (150 µg/ml), tRNA (150 µg/ml) and 5-10×10^6^ cpm of either sense or antisense *suffix* probes, which were added after 24 h of pre-hybridization. After hybridization for 48 h, the membrane was washed twice in 2×SSC-0.5% SDS at 50°C for 25 min and once in 0.5×SSC-0.5% SDS at 50°C for 15 min. [^32^P]-labeled run-off 25 and 14 nt long transcripts, synthesized by T7 RNA polymerase as described above using pGEM-1 DNA digested with SmaI or EcoRI, were used as the size markers. For partial degradation of the 25 nt RNA, treatment with 80 mM NaHCO_3_, 160 mM Na_2_CO_3_ at 60°C for 40 min was performed. The RNA content in each lane was tested by hybridization with a probe prepared by extension of the primer 5′ CAGCATGGACTGCGATATGCGTTC 3′ by AMV RT on 5.8S ribosomal RNA.

### The detection of small RNAs via an SI nuclease protection assay

86 nt long [^32^P]-labeled antisense RNA, corresponding to the 5′ region of *suffix*, was synthesized in 20 µl of a solution containing 1µg of DNA template, 40 mM Tris-HCl (pH 7.5), 6 mM MgCl_2_, 2 mM spermidine, 10 mM NaCl, 10 mM DTT, 1 u/µl RNasin, ATP, GTP, CTP (500 mM each), 1.25 µM [α-^32^P] UTP (6000 Ci/mmol, EIMB), 10 µM unlabelled UTP and 20 u of T7 RNA polymerase (Fermentas). The RNA was gel purified to remove shorter fragments. About 5 µg of the total *Drosophila* RNA preparation was hybridized at 50°C for 12 h in 20 µl of solution containing 0.7 M NaCl, 0.1 M Tris-HCl (pH 7.4), 0.1% SDS and 10^5^ cpm of the RNA probe. After hybridization, 2 µl aliquots were mixed with 10 µl of solution containing 50 mM sodium acetate (pH 4.5), 0.28 M NaCl, 4.5 mM ZnSO4 and 0.1–0.8 u/µl SI nuclease (Promega). SI digestion was performed for 30 min at 20°C, followed by the addition of a 10 µl solution containing 90% formamide, 20 mM EDTA and dyes. The probes were separated in 12% denaturing, 0.2 mm, polyacrylamide gels.

### Detection of transcripts by *in situ* hybridization

Strand-specific DIG-labeled RNA probes were transcribed by T7 RNA polymerase. About 1 µg of DNA template was used in a 20 µl transcription reaction mixture as described above, but now containing ATP, CTP, CTP (1mM each), 0.65 mM UTP and 0.35 mM DIG-11-UTP (Roche). These RNA probes were finally dissolved in 20 µl of water and 80 µl of hybridization solution (HS), containing 50% formamide, 5×SSC, 0.1% Tween 20, 200 mg/ml sheared and denatured salmon DNA and 50 µg/ml heparin. Testes or ovaries were dissected in phosphate-buffered saline (PBS), fixed for 20 min in 4% paraformaldehyde in PBS, washed three times for 5 min in PBT (PBS/0.1% Tween 20), treated with a solution of 50 µg of proteinase K/ml in PBS (5–8 min for testis and 12 min for ovaries), washed with a solution containing 2 mg/ml of glycine in PBT for 2 min and twice for 5 min in PBT, refixed for 20 min in 4% paraformaldehyde in PBS, and washed twice again for 5 min in PBT. After prehybridization in HS at 60°C for 3 to 5 h, the samples were hybridized overnight at 60°C in 300 to 400 µl of HS containing 1 µg of DIG-labeled RNA.

After hybridization, samples were washed three times for 30 min in HS at 60°C, 15 min in 50% HS in PBT at 60°C, twice for 15 min in 2×SSC/0.1% Tween 20 at 60°C, twice for 15 min in 0.2×SSC–0.1% Tween 20 at 60°C, and twice for 15 min in PBT at room temperature. The samples were then incubated for 1–2 h in PBS/0.3% Triton X-100, followed by incubation for 1h in PBS/0.3% Triton X-100/3% goat serum for blocking and in the same solution with anti-DIG-alkaline phosphatase antibodies (Roche, 1∶2000) for 1 h. Finally, samples were washed five times for 15 min in the blocking solution and once for 15 min in PBT. For staining reactions, samples were washed for 10 min in alkaline phosphatase buffer, containing 100 mM NaCl, 50 mM MgCl_2_, 100 mM Tris, pH 9.5, 0.1% Tween 20, and incubated with 1 ml of the buffer containing 20 µl of nitroblue tetrazolium–5-bromo-4-chloro-3-indolylphosphate (NBT/BCIP) stock solution (Roche). Development of the reaction was observed visually under the microscope, and the reaction was usually stopped after 0.5 to 1 h. Samples were then washed five times for 3 min with PBT and mounted in 60% glycerol in PBS.

### 3′RACE

For the cloning of 3′ ends of F element transcripts lacking *suffix* and poly(A) sequences, the addition of poly(A) stretches was performed with the aid of yeast poly(A) polymerase (see the scheme in Figure 7). 10 µg of total poly(A)^−^RNA isolated from pupae or from ovaries was incubated for 10 min at 30°C in a solution containing 25 mM Tris-HCl (pH 7.0), 40 mM KCl, 0.5 mM MnCl_2_, 0.05 mM EDTA, 0.5 mM DTT, 0.2 mg/ml BSA, 10% glycerol, 3.3 µM [α-^32^P]ATP (6000 Ci/mmol, EIMB), 0.5 mM ATP and poly(A) polymerase (USB). After ethanol precipitation, the sample was used for reverse transcription with an oligo(dT)_20_ primer. This was followed by a 100 cycle PCR amplification in a solution containing 10 mM Tris-HCl (pH 8.3); 50 mM KCl; 2 mM MgCl_2_; 0.01% gelatin w/v; 1 mM dNTPs; 1 µg of oligo(dT)_20_ primer; 1 µg of a specific primer with an artificial EcoRI site; 1 u of Taq polymerase and 1 u of Tth polymerase. Amplification conditions were 90°C melting, 37°C annealing and 72°C for extension, for 1 min each. The specific primer, 5′ GAGCACAATCAAAGATTCTGAGAACCATCA 3′, corresponds to the region located about 120 bp upstream of *suffix* in the F element. The cloning was performed using an EcoRI-SmaI digested pUC12 vector. For colony hybridization, an F element specific oligonucleotide, corresponding to the region located about 80 bp upstream of *suffix*, and *suffix*-specific probes were used. The clones hybridizing only with the F element probe were selected and sequenced.
